# Improved Processability and Antioxidant Behavior of Poly(3-hydroxybutyrate) in Presence of Ferulic Acid-Based Additives

**DOI:** 10.3390/bioengineering9030100

**Published:** 2022-02-28

**Authors:** Lionel F. Longé, Laurent Michely, Antoine Gallos, Agustin Rios De Anda, Henri Vahabi, Estelle Renard, Michel Latroche, Florent Allais, Valérie Langlois

**Affiliations:** 1 URD Agro-Biotechnologies Industrielles (ABI), CEBB, AgroParisTech, F-51110 Pomacle, France; lionel.longe@orange.fr (L.F.L.); florent.allais@agroparistech.fr (F.A.); 2 Université Paris Est Creteil, CNRS, ICMPE, F-94010 Creteil, France; michely@icmpe.cnrs.fr (L.M.); agustin.rios-de-anda@u-pec.fr (A.R.D.A.); e.renard@u-pec.fr (E.R.); latroche@icmpe.cnrs.fr (M.L.); 3 Université de Lorraine, Centrale Supélec, LMOPS, F-57000 Metz, France; henri.vahabi@univ-lorraine.fr

**Keywords:** PHB, polyhydroxybutyrate, biopolymer, plasticizer, ferulic acid

## Abstract

Poly(3-hydroxybutyrate), PHB, has gathered a lot of attention for its promising properties—in particular its biobased nature and high biodegradability. Although PHB is prime candidate for the packaging industry, the applications are still limited by a narrow processing window and thermal degradation during melt processing. In this work, three novel additives based on ferulic acid esterified with butanediol, pentanediol, and glycerol (BDF, PDF, and GTF, respectively) were used as plasticizers and antioxidative additives to improve mechanical properties of PHB. Elongation at break up to 270% was obtained in presence of BDF and the processing window was improved nearly 10-fold. The Pawley method was used to identify the monoclinic space group P2 of the BDF. The estimated crystallite size (71 nm) agrees with a crystalline additive. With PHB_70_BDF_30_ blends, even higher elongations at break were obtained though dwindled with time. However, these properties could be recovered after thermal treatment. The high thermal stability of this additive leads to an increase in the fire retardancy property of the material, and the phenolic structure induced antioxidant properties to the samples as demonstrated by radical scavenging tests, further highlighting the possibilities of the PHB/additive blends for packaging applications.

## 1. Introduction

Since their production method was streamlined a few decades ago, poly(3-hydroxyalkanoate)s (PHAs), which are produced by a fermentation process, have shown a growing amount of interest [1,2,3]. Indeed, PHAs are one of the most promising renewable biocompatible and biodegradable polyesters [4,5,6,7,8]. Produced by microorganisms in presence of natural substrates, they offer an excellent alternative to petroleum-based plastics. Poly(3-hydroxybutyrate), PHB, is one of the simplest members of the PHAs family. PHB is a linear semi-crystalline isotactic polyester with high crystallinity, high melting temperature, and excellent resistance to solvents. Similarly, to other PHAs, PHB exhibits complete biodegradability [9,10,11,12,13,14]. However, its industrial applications are still limited today due to its high brittleness, low value for elongation at break and thermal degradation during melt processing. The short permanence above melting point induces degradation that involves a cis-elimination reaction and the formation of crotonic acid [15]. To improve PHB thermal and mechanical properties, a lot of efforts have been put to find different solutions such as formulations with additives [16]. A common workaround is to synthesize copolyesters of PHB with other 3-hydroxyalkanoates moieties, such as 3-hydroxyvalerate and 3-hydroxyhexanoate [17]. Poly(3-hydroxyvalerate) (PHBHV) is the most common co-monomer for industrial application and the resulting copolymer, PHBHV, exhibits significant improvement in the elongation at break depending on monomers ratio. Internal plasticization of PHB have been developed using the bacterial fermentation of copolymers.

On the other hand, the external plasticization is an efficient and rapid method to improve some mechanical properties of polymers. Additives as plasticizers, blend partners, fillers, and crosslinkers may also affect the processing and mechanical properties [18]. Blending PHB with plasticizers may offer the advantage to improve the processability. Plasticizing additives for PHB are diverse, and include aromatic compounds, fatty acids, alcohols, ester, and polymers themselves [19,20,21,22,23,24,25,26,27,28,29,30]. Vegetable oils and their derivatives have also been extensively studied as promising additives into PHA formulations [31,32,33,34]. Semi-interpenetrated networks in which PHB is embedded in network based on sunflower oil and trithiol exhibited a toughening improvement of this polyester [33]. Plasticized PHB formulations were recently prepared with monoterpenes such as linalool, geraniol, and geranyl acetate [34]. Blending with terpenes leads to a decrease of the glass transition temperature (*T_g_*) values and a remarkable increase in the elongation at break combined with a decrease in Young’s modulus with regard to pure PHB. The effect is more pronounced with geranyl acetate thanks to the presence of the segment bearing ester group that increases free volume and molecular mobility. However, there are still challenges to be overcome to open the new applications to PHB. Ferulic acid is a biobased compound that can be extracted though different processes from lignocellulosic biomass. Due to the various functional groups, it can be easily functionalized and its phenol group usually carry on antioxidant properties even after functionalization. Ferulic acid derivatives were recently found to have plasticizing effects on polylactic acid (PLA) and polycaprolactone (PCL) [35,36,37].

The objective of this study is to develop the appropriate plasticizer for improving mechanical properties. We aim at preparing plasticized PHB with ferulic derivatives. The bis-O-dihydroferuloyl 1,4-butanediol (BDF) was blended with PHB to have a direct comparison with the observed plasticizing effect of BDF in PLA. Two others ferulic acid derivatives (bis-O-dihydroferuloyl 1,5-pentanediol, and tris-O-dihydroferuloyl glycerol), hereafter named PDF and GTF, were also blended with PHB to study the effect of various molecular designs. PHB was melt-blended with BDF, PDF, and GTF using extrusion process. The influence of the chemical structure of these compounds on the thermal properties, mechanical properties, dynamic mechanical properties, thermal stability, and flammability behavior of PHB was studied. A special attention was paid to the evolution of the mechanical properties and recrystallization of the blends over time.

## 2. Materials and Methods

PHB powder (Batch T19) without any plasticizer was kindly supplied by BIOMER, Schwalbach am Taunus, Germany (Mw = 230,000 g·mol^−1^). Ferulic acid was acquired from Biosynth-Carbosynth, Eching, Germany. Palladium (10%) on activated charcoal, 2,2-diphenyl-1-picrylhydrazyl and sodium chloride were purchased from Fisher Scientific, Illkirch, France. Hydrochloric acid (37%), anhydrous magnesium sulphate, sodium bicarbonate, celite, acetone, ethanol, methanol, dichloromethane, and ethyl acetate were obtained from VWR, Fontenay-sous-Bois, France. Ferulic acid (≥99% purity) was purchased from Sigma-Aldrich. Butane-1,4-diol, pentane-1,5-diol and glycerol were acquired from Alfa Aesar, Kandel, Germany. Solid supported Cal-B (Novozyme 435) was procured from Novozymes, Le Pecq, France. Deuterated acetone and deuterated chloroform were ordered from Eurisotop, Saint-Aubin, France.

### 2.1. Synthesis of Ferulate Derivatives

Synthesis of the different additives was performed in three steps. First, in a round bottom flask, ferulic acid (150 g, 0.77 mol) was dissolved in large excess of ethanol (600 mL). The mixture is vigorously stirred, then concentrated hydrochloric acid (37% *w*/*w*, 8.8 mL, 0.12 mol), was added into the solution under constant stirring. The reaction was allowed to proceed at reflux overnight. The mixture is then allowed to cool down and ethanol is removed under reduced pressure and replaced by ethyl acetate (600 mL). The organic phase is then washed three times with saturated sodium carbonate solution (3 × 100 mL) and once with brine solution (100 mL). The organic layer is then recovered and dried over anhydrous magnesium sulphate. An aliquot of the solution was taken, and ethyl acetate was removed under reduced pressure to yield ethyl ferulate. Purity was measured higher than 99% by NMR and yield was calculated to be 98.0% (168.2 g, 0.76 mol). Secondly, the round bottom flask containing the ethyl ferulate (168.2 g, 0.76 mol) solution was sealed with a septum and nitrogen was bubbled through for 15 min. Palladium on activated charcoal (8.4 g) was quickly added to the flask then nitrogen was further bubbled for 5 min. Dihydrogen was then flushed constantly in the round bottom flask, under constant gentle stirring, until hydrogenation was completed (ca. 48 h). The mixture is then filtered over celite, and ethyl acetate was removed under reduced pressure to yield hydrogenated ethyl ferulate (163.8 g, 0.73 mol), 96.5% yield, >99.9% purity. Finally, hydrogenated ethyl ferulate (2) (163.8 g, 0.73 mol) and butane-1,4-diol (21 mL, 0.24 mol) were vigorously mixed in a round bottom flask. Solid supported Cal-B (16.3 g) was poured in the mixture under constant stirring. Heating was set to 75 °C and the round bottom flask was connected to high vacuum pump and left overnight with gentle stirring. After being allowed to cool down, acetone was used to dissolve and filter the mixture to remove solid supported Cal-B. Acetone was then removed under reduced pressure to yield a white solid. Recrystallization in ethanol (500 mL) yielded BDF (67.86 g, 0.15 mol), 69.1% yield, 97.2% purity. Yield can be greatly increased by further recrystallization. For PDF synthesis, similar reaction was performed with pentanediol instead of butanediol. Hydrogenated ethyl ferulate (150 g, 0.67 mol) and pentane-1,5-diol (23 mL, 0.22 mol) were added to a round bottom flash and vigorously stirred until complete homogenization. Solid supported Cal-B (15.0 g) was added in the mixture under constant stirring. Heating was set to 75 °C and the round bottom flask was connected to high vacuum pump and left overnight with gentle stirring. After being allowed to cool down, acetone was used to dissolve and filter the mixture to remove solid supported Cal-B. Acetone was then removed under reduced pressure to yield a yellow oil. Purification was easily done by flash chromatography. The product was loaded on silica with acetone and the following solvent profile was applied to purify the mixture: 5 column volumes (CV) with a 95/5 cyclohexane/isopropanol, then increase to 80/20 during another 5CV. PDF can be collected during the second phase of the purification, while some remaining ethyl ferulate can be collected during the first phase. Total yield for PDF 84.1% (85.19 g, 0.19 mol), purity > 99%.

### 2.2. Extrusion Process

Extrusion of polymer-additives formulation was performed on a compounding extruder HAAKE™ MiniLab II twin screw, screw diameter 16 mm, 24 mm. Screws are set in co-rotation, at 60 rpm. Extrusion temperature was set to 170 °C. HAAKE MiniJet Pro piston injection molding system was used for the injection molding of sample specimens. DMA test bar mold was used, with dimensions of 60 × 10 × 1 mm. The mold was maintained at 45 °C during injection.

### 2.3. Characterization

Tensile specimens were prepared using a cutting die mounted on an arbor press. Specimen were prepared according to ISO 527-2 specification, type 5B. Traction tests were performed on an INSTRON 5965 dual column tabletop testing systems set to tensile testing. The equipment was fitted with 2530 series static load cells, 100 N or 2 kN when required. Strain rate was set to 1 mm/min. Tests were performed in triplicate at room temperature. Dynamic mechanical analyses (DMA) were conducted on a TA Instrument DMA Q800 in tension deformation mode. Frequency was set to 1 Hz and strain to 0.06%. Temperature was raised from −140 °C to 170 °C at a ramp of 3 °C/min. The samples had dimensions (20 × 5 × 0.92 mm) and all tests were done under air. The storage modulus (E′), loss modulus (E″), and loss factor (tan δ) of each specimen were obtained as a function of temperature. X-ray diffraction (XRD) was performed using a Bruker D8 advance diffractometer (Cu-Kα radiation), in the 2θ-range from 7 to 55° with a step size of 0.02°. Crystallographic properties of the patterns were analyzed by the Pawley and Rietveld methods using TOPAS 4 [38]. Powder density was obtained by a manometric method with an ULTRAPYC 1200e helium pycnometer from Quantachrome.

Differential scanning calorimetry (DSC) measurements were recorded on a TA Instruments DSC25. A first heating run from −80 °C to 200 °C with a heating rate of 10 °C/min was performed to determine the melting temperature (*T*_m_) and the fusion enthalpy (Δ*H*_m_). This was followed by a cooling run to −80 °C with a cooling ramp of 200 °C/min. The *T_g_* was obtained in a second heating run from −80 °C to 200 °C at 10 °C/min. The degree of crystallinity χ_c_ may be calculated via the total enthalpy method according to Equation (1)

(1) $$\chi_{c}=\frac{\Delta H_{m}}{W_{\mathrm{PHB}}\Delta H{{}^{\circ}}_{m}}\times 100$$

where Δ*H*_m_ is the specific enthalpy of melting of the sample studied, WPHB the weight fraction of the PHB in the blend and Δ*H*_m_ represents the specific enthalpy of melting for the 100% crystalline PHB, taken as 146 J/g. The degree of crystallinity *χ_c_* was calculated as a function of the real amount of PHB in each sample.

### 2.4. Antioxydant Activity and Flammability Measurements

Radical scavenging activity (RSA) was measured by simple test with 2,2-diphenyl-1-picrylhydrazyl (DPPH). A solution of DPPH at 0.1 mM in ethanol was prepared fresh and kept away from light. A sample of the polymer blend was then placed in a test tube along with 3 mL of the DPPH solution (immersion length 3.2 cm, i.e., total surface in contact with solution 7.14 cm^2^). After one hour in total darkness and at room temperature the absorption at 515 nm was measured and compared to the absorption of the original solution. The radical scavenger ability (*RSA*) was then calculated as in Equation (2)

(2) $$RSA\;\left(\%\right)=\frac{Abs_{reference}-Abs_{sample}}{Abs_{reference}}\times 100$$

where *Abs* reference is the absorbance of the 0.1 mM of DPPH solution without a sample and *Abs* sample is the absorbance of the 0.1 mM solution of DPPH with the polymer sample.

A pyrolysis combustion flow calorimeter analysis (PCFC) apparatus from Fire Testing Technology (FTT) Company-UK, was used to evaluate the flammability behavior of samples. A small quantity of samples, between 2 and 4 mg, were pyrolyzed up to 750 °C. The heating rate was fixed at 1 °C/s. Then, gases obtained from sample pyrolysis were collected and transferred into another compartment for combustion at 900 °C containing 20% of oxygen. Huggett’s relation (1 kg of consumed oxygen corresponds to 13.1 MJ of released energy) allowed obtaining of the most important parameters related to flammability: peak of heat release rate (pHRR), temperature at pHRR (TpHRR), and total heat release (THR). For each sample three tests were performed, and the related accuracy was estimated around 5%.

## 3. Results and Discussion

### 3.1. Synthesis of the Additives Based on Ferulic Acid

**Figure 1:**
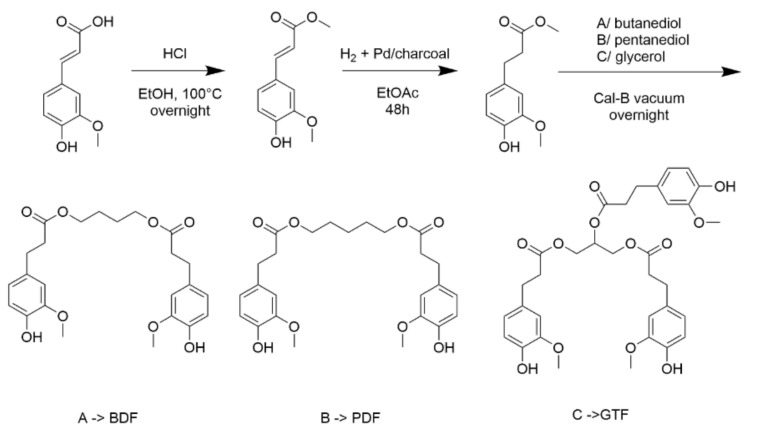
Synthesis of BDF, PDF, and GTF additives from ferulic acid.

Three esters have been synthesized from ferulic acid (Figure 1). The first step is an esterification of ferulic acid in presence of ethanol, followed by the hydrogenation of the non-aromatic double bond. The transesterification in the presence of butanediol, pentanediol or glycerol is then catalyzed by solid supported Cal-B to obtain ferulic derivatives called butanediol diferulate, BDF (A), pentanediol diferulate, PDF (B), and glycerol triferulate GTF (C) respectively. BDF and PDF are diesters while GTF, obtained in presence of glycerol is the triester of ferulic acid. Physical properties of BDF, GTF, and PDF are reported in Table 1.

The three compounds exhibit a glass transition temperature which was attributed to some medium-long range order in the material as the compound form a non-covalent oligomer by π-stacking of the aromatic rings. As expected, the longer five carbon chain in PDF decreases the *T_g_* compared to BDF which has only four carbon chain. Yet, that one carbon difference is also responsible for a completely different crystallization behavior. This seems sufficient to have different intermolecular interactions (polar interaction between the ester groups, aromatic groups, hydroxyl functions, etc.) that could explain this difference between BDF and PDF. BDF has a *T*_m_ at 110 °C while analysis by DSC could not detect any italic>T*T*_m_ for PDF, suggesting different intermolecular interactions for those two compounds. GTF, being synthesized around a short triol, displays a much higher *T_g_* at 42 °C due to the presence of the three aromatic rings that stiffens the system. We then observed an exudation of the PDF on the surface of our samples during their storage. This observation allowed us not to retain PDF for the rest of our study because the blends are not stable.

### 3.2. PHB/Additives Blends and Their Mechanical Properties

The major drawback of PHB during hot melt extrusion is that the melting point and the extrusion temperature are very close to the degradation temperature. Therefore, if PHB residence time in the extruder is prolonged, temperature and shear stress will start to degrade the polymer, causing oxidation (i.e., seen as samples browning), distinctive odor release attributed to crotonic acid and loss of mechanical properties. To overcome this inconvenient, PHB and the ferulic acid-based additives were combined by hot melt extrusion in a twin-screw mini-extruder and injected directly into a mold. By using a mini-extruder, PHB residence time is diminished, which limits the polymer degradations. Different formulations of PHB_x_BDF_y_ blends were prepared to study the influence of BDF concentrations (Table 2).

**Figure 2:**
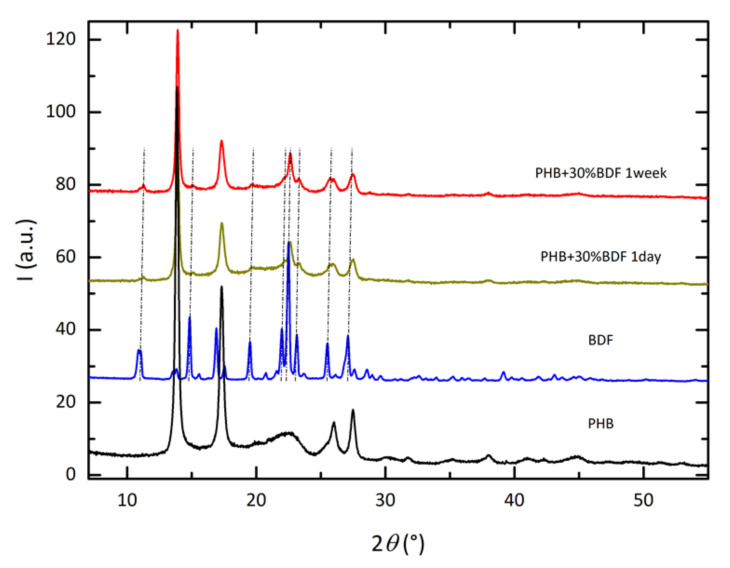
X-ray diffraction patterns of (from bottom to top) PHB (black), BDF (blue), PHB_70_BDF_30_ as-prepared (one day), and PHB_70_BDF_30_ after one week. Vertical dotted lines added for BDF are a guide for the eyes.

Theoretical glass transition temperature of BDF (−17.7 °C) matches quite closely with the experimental *T_g_* measured by DSC (−19 °C). Furthermore, the measured *T_g_* for the PHB_x_/BDF_100−x_ blends also line up closely to the theoretical values determined by the Fox equation when the blends contain less than 30 wt % of BDF. The crystallinity of the PHB is nearly unchanged whatever the content of BDF, as shown by the values of the melting enthalpies in the different blends. Even if the BDF concentrations above 20% start to hinder PHB crystallization, the BDF interference with PHB is only limited to a shift in melting point *T*_m_ from 177 °C to 167 °C. This decrease of *T*_m_ is certainly due to the decrease of crystallite sizes as the total amount of crystalline PHB part remains constant. The diffraction pattern of PHB is shown in Figure 2. The phase is crystalline though a broadening of the diffraction peaks is observed in the range 20–25° in 2θ. The diffraction pattern was indexed with the orthorhombic cell proposed by Kawaguchi and Doi [39]. The structure was refined with the atomic positions given by Brückner et al. [40] in space group P212121. The cell parameters obtained from the Rietveld and Loopstra method [41] are given in Table 3. The estimated crystallite size (286 nm) agrees with a semi-crystalline material. For the X-ray pattern of BDF, the phase is crystalline though the diffraction peaks are relatively broad. The Pawley method [42] was used to determine the space group and cell parameters of BDF. The diffraction lines can be fully indexed with a monoclinic space group P2 and the cell parameters are given in Table 3.

The estimated crystallite size (71 nm) agrees with a crystalline material. The calculated density (1.282) of BDF deduced from the chemical formula C_24_H_30_O_8_ (M = 446.5 g) and the cell volume obtained by the Pawley method (V = 1156.7 Å^3^) is in good agreement with the measured one (1.277), assuming Z = 2. The diffraction pattern of the PHB_70_/BDF_30_ is also shown as prepared (1 day) in Figure 2. The pattern can be described by a combination of the two single phases BDF and PHB, though BDF looks much less crystalline than the pristine material. All diffraction peaks can be accounted, and the sample can be seen as a physical mixture of the two phases without significant chemical reactions between them. It can be stated that introducing 30% BDF lowers the melting temperature of PHB by 10 °C, which allows to decrease the processing temperature of the samples by extrusion. It is possible that BDF not only acts as a chain spacer in the amorphous phase of PHB but plays the same role during the melting and extrusion process. By spacing the chains, BDF can reduce the polymers viscosity and melting point, leading to lower overall processing temperatures. This is beneficial as the heat extrusion effect on the polymer degradation is reduced. For the remaining of the investigation, a concentration of 30% of additive in the blends was considered.

**Figure 3:**
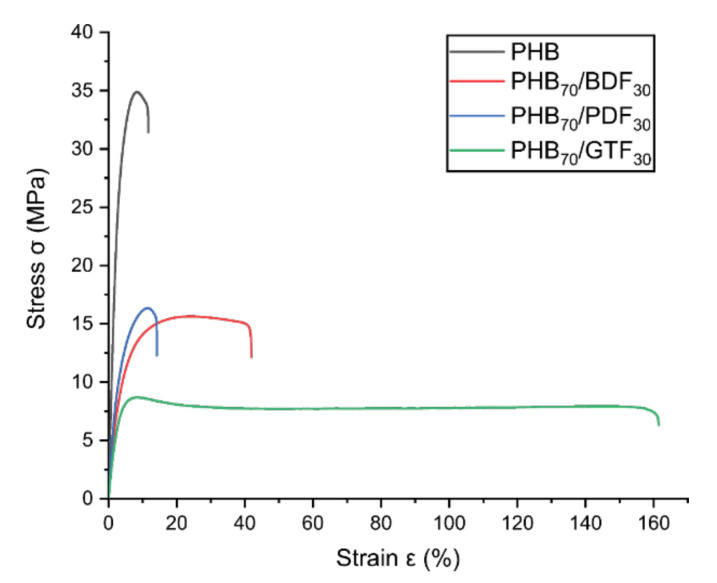
Strain–stress curve of PHB/additive samples, three hours after extrusion: PHB_70_BDF_30_, PHB_70_PDF_30_, PHB_70_GTF_30_, and pure PHB.

Mechanical properties showed that the incorporation of the ferulic acid-based additives induces a substantial increase in the elongation at break of the material which was measured three hours after extrusion in the case of PHB_70_GTF_30_ and PHB_70_BDF_30_. For PHB_70_GTF_30_, it jumps from 11% for pure PHB to 178% (Figure 3, Table 4). This increase is paralleled by a decline of the Young’s modulus E and of the stress at break σr. Indeed, σr decreases from 29.7 for pure PHB to 5.8 MPa for PHB_70_GTF_30_. This means that both GTF and BDF act also as mechanical plasticizers, yielding toughened PHB formulations. For PHB_70_PDF_30_, no increase of the elongation at break could be detected compared to pure PHB. Additionally, it was found that during storage at room temperature, some oily substance exuded from the samples. Unlike BDF and GTF, PDF is a liquid at room temperature which explains that this behavior is not observed for these compounds. For those reasons, PDF could not be considered as a good candidate for improving the toughening mechanical properties of PHB or for medium-long term packaging applications contrary to BDF and GTF.

### 3.3. Evolution of Mechanical Properties over Time

**Figure 4:**
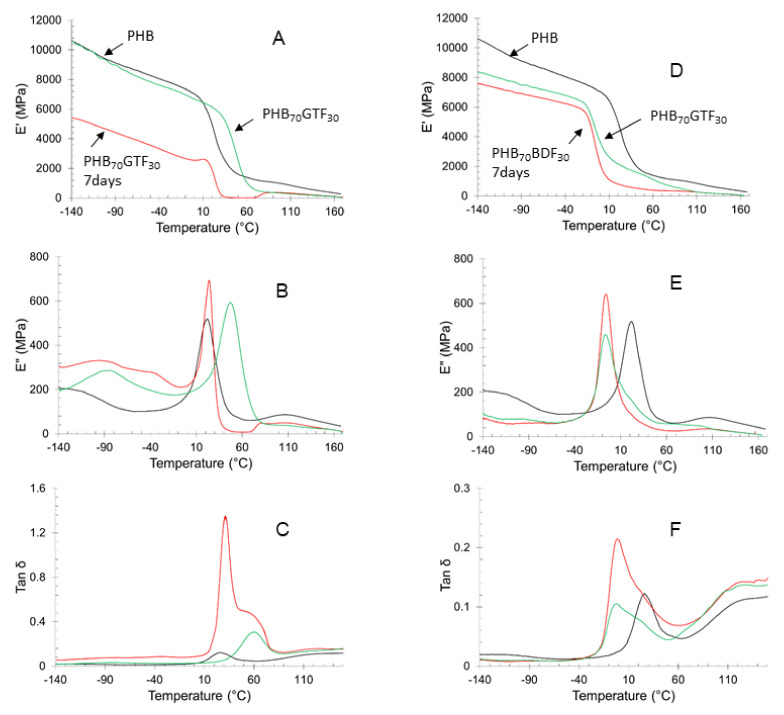
DMA analysis. (**A**) Storage modulus, (**B**) loss modulus, and (**C**) tan delta of PHB after extrusion (in dark), PHB_70_GTF_30_ ½ h after extrusion (in red), PHB_70_GTF_30_ 7 days after extrusion (in green). (**D**) Storage modulus, (**E**) loss modulus, and (**F**) tan delta of PHB after extrusion (in dark), PHB_70_BDF_30_ 0.5 h after extrusion (in red), PHB_70_BDF_30_ 7 days after extrusion (in green).

PHB mechanical properties are known to quickly evolve with time after solidifying. After three hours, PHB properties are usually mostly settled but can still slightly evolve. By blending additives into PHB, the phenomenon kinetics might vary. As such, the blends mechanical properties were monitored by DMA over a period of one week for PHB_70_BDF_30_ and PHB_70_GTF_30_ (Table 5, Figure 4). Values of tan δ measured by DMA show important changes for PHB-GTF blends for which a sharp decrease in intensity can be noted. Furthermore, the presence of two peaks in the signal can also be detected, one at 30 °C characteristic of PHB, and a second one at 60 °C for GTF. After one week, the material properties changed significantly as only the peak at 60 °C remains. This shift in tan δ peak temperature and intensity is due to an increase of the crystallization ratio of the blend. Indeed, as the chains reorganize and crystallize, the material becomes more brittle (i.e., diminishing tan δ intensity) and the *T_g_* increases (i.e., tan δ peak shifts to higher temperatures). This phenomenon also accounts for the jump in E′ at 40 °C, from 45 to 4510 MPa. However, the hardening of the samples leads to high brittleness. Specimens for traction test could no longer be prepared as samples shattered upon handling. Therefore, it was concluded that GTF was not suitable as an additive for PHB. In the case of PHB_70_BDF_30_ samples, the maximum of tan δ was observed at −1 °C. The peak is also much wider and asymmetrical. The shoulder discerned at higher temperature, around 20 °C, is attributed to the presence of BDF. A reorganization of the material occurs between the two measurements with an overall stiffening of the material as manifested by the increase of E′ at 40 °C from 540 to 1630 MPa. This value is yet close to the initial value for PHB, 2120 MPa.

**Figure 5:**
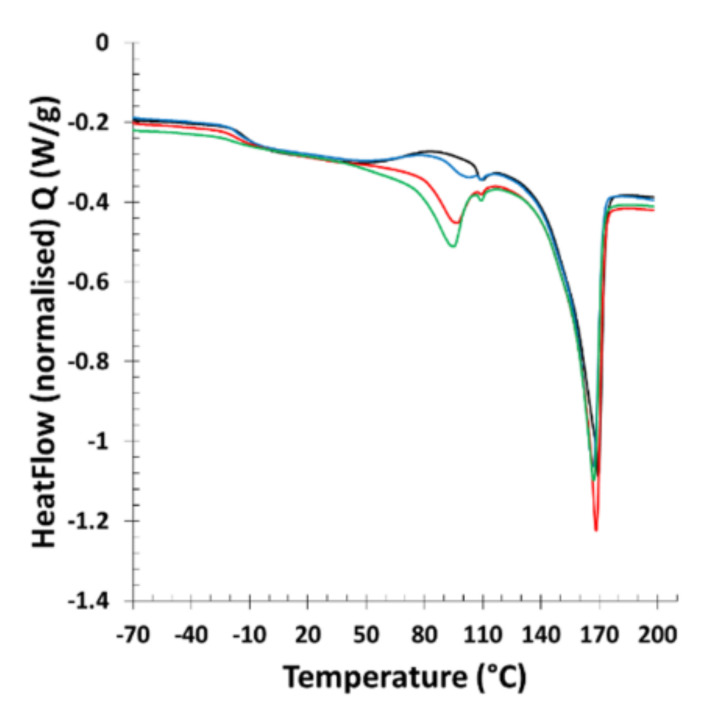
DSC analysis of PHB_70_BDF_30_ after extrusion after 30 min (in dark), PHB_70_BDF_30_ 0.25 day after extrusion (in blue), PHB_70_BDF_30_ one day after extrusion (in red), PHB_70_BDF_30_ seven days after extrusion (in green).

Consequently, the addition of BDF to PHB reduces the stiffening of the material during storage. This behavior is advantageous as PHB alone is stiff and brittle. For better understanding of the BDF and PHB crystallization, their structures were studied by X-ray diffraction. After one week, the diffraction pattern of the PHB_70_BDF_30_ mixture does not show significant evolution with time (Figure 2). The amorphous contribution observed for the as-prepared sample seems however significantly reduced for the one-week sample, indicating some possible recrystallization phenomenon. This fact was confirmed by DSC measurements (Figure 5).

Analysis by DSC over the one-week period of the PHB70BDF30 samples highlights a BDF crystallization after 24 h. This crystallization is responsible for the property changes as they are closely linked to the reorganization of the macromolecular chains of the amorphous into crystalline domains. The BDF crystallizes in the sample over time which causes a change in the mechanical properties of the PHB/BDF blends which become stiffer and much less flexible.

Mechanical tests very quickly after extrusion because we observed a very important flexibility of the PHB samples which contain BDF. It is really very difficult to prevent crystallization of PHB, which has a very fast crystallization rate but the strain at break of PHB_70_BDF_30_ is around 253% although the strain at break of the PHB is around 11% just after extrusion.

**Figure 6:**
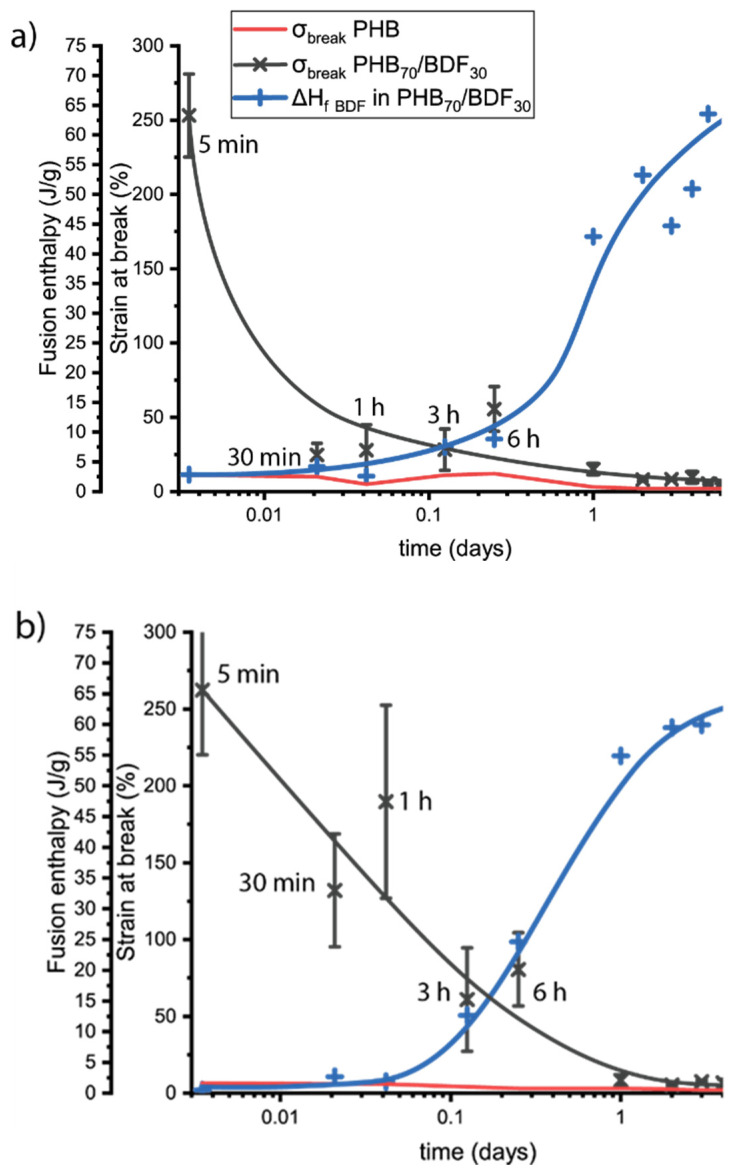
Follow up of polymer deformation at break for PHB (gray) and PHB_70_BDF_30_ (red) (**a**) after extrusion and (**b**) after annealing at 120 °C. Blue dashed line represents the fusion enthalpy of BDF in the PHB_70_BDF_30_.

The evolution of the mechanical properties was therefore followed over time and to see if this phenomenon is reversible, the samples were subjected to thermal treatments at 120 °C, i.e., above the *T*_m_ of BDF but well below the *T*_m_ of PHB. It turns out that three anneals at 120 °C for 5 min were necessary for the sample to recover its initial properties, i.e., an elongation at break of 260%, as shown in Figure 6. Five minutes after extrusion, PHB samples already have low elongation at break around 11%, while for PHB_70_BDF_30_ it is 25 times higher, at 253%. This value decreases rapidly, and after one hour it is already down to 27%. Simultaneously the BDF was observed to crystallize in the sample as the normalized fusion enthalpy increases steadily to a plateau around 65 J/g. Even though the two phenomena seem connected, they do not happen concomitantly. Indeed, the decline in elongation at break occurs marginally before the increase of Δ*H*_m_. After one week, the samples were annealed 5 min at 120 °C. Elongation at break increased by 34%, five times higher than annealed PHB. Following three anneals, properties like the fresh samples could be recovered (Table 6). 262% of elongation at break was measured five minutes after annealing, and 190% after one hour. Compared to the non-annealed samples, the drop in εbreak was about 10 times slower, while, at the same time, the crystallization kinetic was slightly faster.

### 3.4. Antioxidant Properties and Thermal Stability

**Figure 7:**
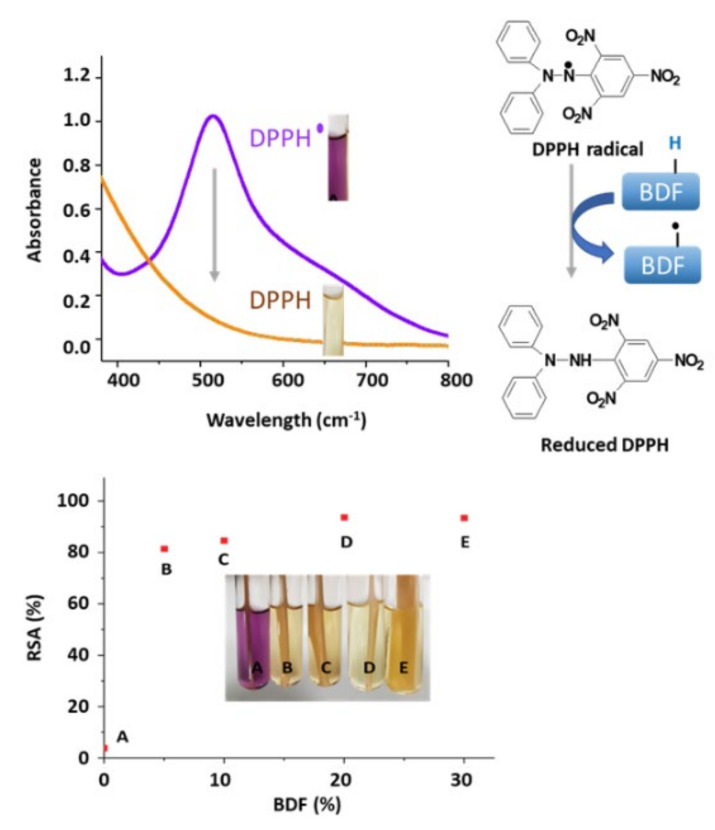
Radical scavenging activity of (**A**) pure PHB, (**B**) PHB_95_BDF_5_, (**C**) PHB_90_BDF_10_, (**D**) PHB_80_BDF_20_, and (**E**) PHB_70_BDF_30_.

BDF is a phenolic compound that exhibits two phenol groups per molecule. As such, some radical-scavenging property is expected. Accordingly, by blending BDF and PHB the resulting material is anticipated to exhibit antioxidant behavior. Radical scavenging activity (RSA) can be measured by reduction of DPPH in solution. The DPPH radical scavenging assay is widely used to assess the antioxidant activity of phenolic compounds [43,44]. At 517 nm, this relaxation and swelling control radical has an absorbance maximum. When this molecule reacts with a hydrogen donor such as an antioxidant, the absorbance decreases and a change in color of the solution from purple to yellow is observed. As shown in Figure 7, concentration as low as 5%*w*/*w* was found to reduce 81% of DPPH in solution. With 20% and above, 94% radical scavenging activity can be obtained. This behavior makes BDF a promising additive for applications where antioxidant properties are sought after.

**Figure 8:**
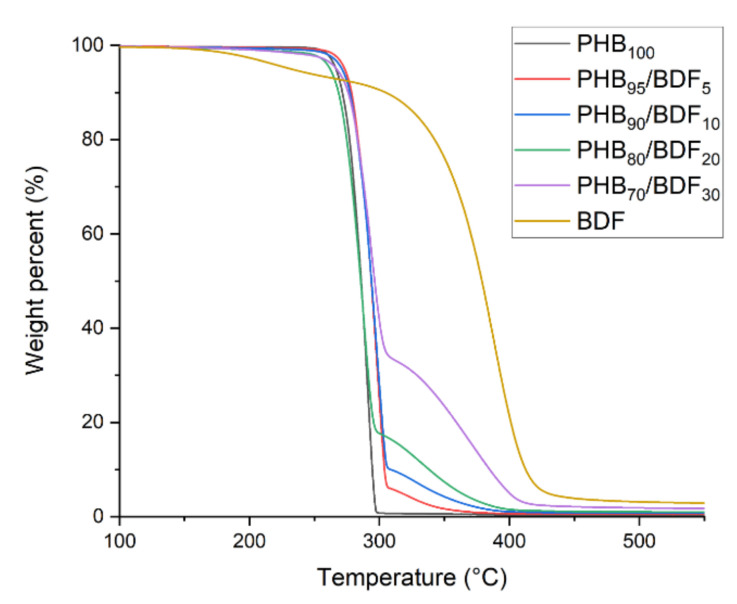
Thermal stabilities of different PHB-BDF blends measured by TGA.

**Figure 9:**
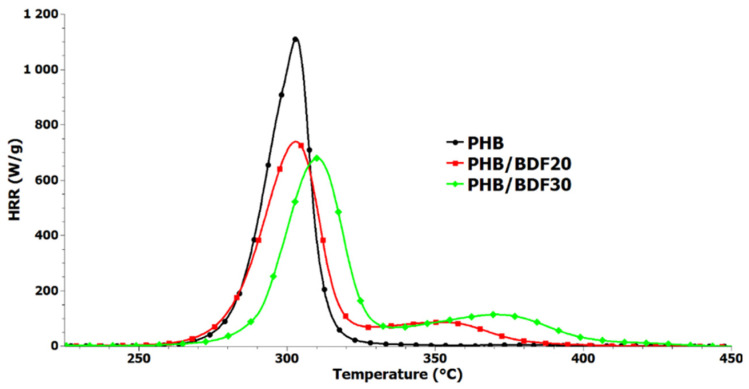
Heat release rate (HRR) curves for PHB, PHB_80_BDF_20_, and PHB_70_BDF_30_ obtained from pyrolysis combustion flow calorimeter analysis (PCFC) tests.

The TGA shows the loss of mass as a function of temperature. This loss of mass is due to the loss of volatile fragments following the important degradations of polymer chains leading to small molecules. On Figure 8 the temperature at which 20% mass loss is observed is 280 °C for PHB and 360 °C for BDF. The introduction between 5% and 40% of BDF increases the thermal stability by 10 °C and the e20% mass loss is now observed at 290 °C. Pyrolysis combustion flow calorimetry (PCFC) tests were performed according to ASTM D7309 to evaluate the flammability of PHB, PHB_80_BDF_20_ and PHB_70_BDF_30_ samples [45]. Figure 9 displays the curves of heat release rate as a function of temperature and several parameters extracted from these curves, including peak of heat release rate (pHRR), total heat release (THR), and temperature at pHRR, summarized in Table 7. Pure PHB shows a highly flammable character demonstrated by an intense pHRR 1074 W/g at 305 °C. The presence of BDF in PHB significantly changed the flammability behavior of PHB. The slope of HRR curves were decreased by increasing the percentage of BDF. Moreover, the pHRR was meaningfully decreased and reached 723 W/g and 659 W/g for PHB_80_BDF_20_ and PHB_70_BDF_30_, respectively. Furthermore, the temperature at pHRR was increased from 305 °C for pure PHB to 310 °C for PHB_70_BDF_30_, while for PHB_80_BDF_20_ it remained similar that of pure PHB, at 305.5 °C. It was also noticed that there is quite no change in THR for samples containing BDF compared to pure PHB.

## 4. Conclusions

Among the three ferulic acid derivatives, only BDF and GTF showed a plasticizing effect of PHB with a clear increase in elongation at break unlike PDF. However, the mechanical properties of the mixtures vary with time due to the crystallization of the additives. This effect is very important in the case of GTF because the specimens become so brittle after one week that it is no longer possible to handle them. The BDF is very pro-mising because it increases the elongation at break from 11% for PHB to 260% for the PHB_70_BDF_30_ blend. Although this value is not stable over time, it is possible to recover this property after a short five-minute heat treatment of PHB at a temperature much lower than the melting temperature of PHB but just above the melting temperature of BDF. For this purpose, an X-ray diffraction study has allowed for the first time to specify the crystal structure and the lattice parameters of BDF. This derivative which had already shown its properties as plasticizer for PCL and PLA confirms here all its interest during the formulation of PHB. The reversibility of the mechanical properties according to the temperature combine with the antioxidant properties and the resistance to the flammability will undoubtedly allow to consider new applications for the PHB. The incorporation of BDF decrease the hydrophobicity of PHB surface as it was shown by the values of contact angle with water from 70° to 63.5° in presence of BDF. The effect of ferulic acid-based derivatives on the kinetics of enzymatic and hydrolytic degradations required a further study to precise the long-term behavior of these biodegradable polyesters.

## Figures and Tables

**Table 1:** Molar masses and thermal properties of ferulic acid derivatives determined by DSC.

	M (g·mol^−1^)	*T_g_* (°C)	*T*_m_ (°C)	Δ*H*_m_ (J/g)
BDF	446.2	−19	112	108
PDF	460.2	−22	-	-
GTF	626.2	+42	-	-

**Table 2:** Formulations and thermal properties of different PHBxBDF100-x blends determined by DSC after different times since the extrusion process.

PHB_x_BDF_100−x_	Time *_after extrusion_* (day)	*T_g theo._* (°C)	*T_g_* (°C)	*T*_m__PHB_ (°C)	Δ*H_m_*_PHB_ (J/g)	*T*_m__BDF_ (°C)	Δ*H*_m__BDF_ (J/g)
PHB_100_BDF_0_	7	-	+0.9	177	101	-	-
PHB_95_BDF_5_	7	−0.2 ^b^	−0.7	173	98	-	-
PHB_90_BDF_10_	7	−1.2 ^b^	−1.5	172	99	-	-
PHB_80_BDF_20_	7	−3.2 ^b^	−4.1	168	98	100	9
PHB_70_BDF_30_	0.25	−5.3 ^b^	−17	167	93	102	9
PHB_70_BDF_30_	1	−5.3 ^b^	−17	168	97	96	37
PHB_70_BDF_30_	7	−5.3 ^b^	−18	167	91	95	56
PHB_0_BDF_100_	7	−17.7 ^a^	−19.0	-	-	112	108

^(a)^ Calculated by *T_g_* = 2/3 *T*_m_. ^(b)^ Determined by Fox equation.

**Table 3:** Crystallographic parameters of BDF and PHB determined by X-ray diffraction (estimated standard deviations (esd) given between brackets apply to the last digits).

Phase	Space Group	a (Å)	b (Å)	c (Å)	β (°)	Crystal Size (nm)
BDF	P2 (N°3)	13.055 (2)	11.029 (2)	8.682 (2)	112.30 (2)	71 (5)
PHB	P2_1_2_1_2_1_	5.713 (2)	13.171 (5)	6.055 (3)	-	286 (49)

**Table 4:** Mechanical properties of PHB and blends three hours after extrusion.

	Young Modulus (MPa)	Stress at Break (MPa)	Elongation at Break (%)
PHB_100_	1169 ± 38	29.7 ± 1.6	11 ± 1.6
PHB_70_BDF_30_	417 ± 38	11.4 ± 1.1	42 ± 13.9
PHB_70_PDF_30_	510 ± 82	12.3 ± 1.7	12 ± 5.5
PHB_70_GTF_30_	211 ± 93	5.8 ± 0.8	178 ± 48.7

**Table 5:** Thermo-mechanical properties of different PHB-additive blends measured by DMA after (a) 3 h and (b) 1 week.

	E′ at 20°C (MPa)	E′ at 40 °C (MPa)	E″ Tα (°C)	E″ Tβ (°C)	Tan δ (°C)
PHB_70_BDF_30_ (a)	820	540	−5		−1
PHB_70_BDF_30_ (b)	2190	1630	−7		−2
PHB_70_GTF_30_ (a)	2000	45	24	−100	31
PHB_70_GTF_30_ (b)	6140	4510	47	−86	60

**Table 6:** Effects of successive anneals at 120 °C on mechanical properties.

Number of Anneals	ε_break_PHB_70_BDF_30_ (%)	ε_break_ PHB_100_(%)	σ_break_PHB_70_BDF_30_ (MPa)	σ_break_ PHB_100_ (MPa)
0	253 ± 20	11 ± 0.2	12.5 ± 1.3	22.8 ± 0.5
1	34 ± 14	6 ± 0.2	17.0 ± 0.4	36.1 ± 1.9
2	54 ± 13	5 ± 0.8	15.2 ± 0.8	36.3 ± 2.8
3	262 ± 42	7 ± 2.0	18.1 ± 1.1	28.8 ± 1.2

**Table 7:** Summary of results obtained in PCFC tests for PHB, PHB_80_BDF_20_, and PHB_70_BDF_30_.

	pHRR (W/g)	T_pHRR_	THR (kJ/g)	Reduction in pHRR (%)
PHB	1074	305	22.3	-
PHB_80_/BDF_20_	723	305.5	22.8	33
PHB_70_/BDF_30_	659	310	23	39

## Data Availability

Not applicable.
